# Elimination of *Plasmodium falciparum* in an area of multi-drug resistance

**DOI:** 10.1186/s12936-015-0838-5

**Published:** 2015-08-16

**Authors:** Khin Maung Lwin, Mallika Imwong, Preyanan Suangkanarat, Atthanee Jeeyapant, Benchawan Vihokhern, Klanarong Wongsaen, Georges Snounou, Lilly Keereecharoen, Nicholas J White, Francois Nosten

**Affiliations:** Shoklo Malaria Research Unit, Mahidol-Oxford Tropical Medicine Research Unit, Faculty of Tropical Medicine, Mahidol University, Mae Sot, Thailand; Centre for Tropical Medicine and Global Health, Nuffield Department of Medicine, University of Oxford, Oxford, UK; Mahidol Oxford Research Unit, Faculty of Tropical Medicine, Mahidol University, Bangkok, Thailand; Department of Molecular Tropical Medicine and Genetics, Faculty of Tropical Medicine, Mahidol University, Bangkok, Thailand; Sorbonne Universités, UPMC Univ Paris 06, UPMC UMRS CR7, 75005 Paris, France; Centre d’Immunologie et de Maladies Infectieuses (CIMI), Paris, Institut National de la Santé et de la Recherche Médicale (Inserm) U1135, Centre National de la Recherche Scientifique (CNRS) ERL 8255, 75013 Paris, France

## Abstract

**Background:**

Resistance to the artemisinin derivatives in *Plasmodium falciparum* has emerged in Cambodia and is now spreading throughout South-East Asia. The rapid elimination of *P. falciparum* seems to be the only viable option to avoid a public health disaster but this is difficult because even in low transmission settings many residents have asymptomatic parasitaemias.

**Methods:**

In response to a large number of malaria cases reported in three remote villages on the Thai-Myanmar border where malaria is endemic and the disease is seasonal, surveys were conducted using an ultra-sensitive qPCR assay (LOD 22 parasites per mL). In one of the villages where it was feasible, mass anti-malarial drug administration was proposed to the population as a potential solution, and this was adopted.

**Results:**

In the three villages 204/356 (57.3 %), 212/385 (55.1 %) and 195/286 (68.2 %) of the resident populations were positive by qPCR (approximately one-third *P. falciparum* and two-thirds *P. vivax)*. Of those positive for *P. falciparum* 62 % carried single point mutations in the *P. falciparum* kelch protein (a marker of artemisinin resistance). In one of the villages 217 of 674 inhabitants received at least one dose of dihydroartemisinin-piperaquine chemoprevention in June 2012, 155 (71.4 %) received two consecutive months, and 98 (45.2 %) received three treatment doses. The chemoprevention was generally well tolerated. The sub-microscopic reservoir of *P. falciparum* malaria was eliminated during the six-month follow-up period (prevalence fell from 7 to 0 %); *P. vivax* malaria persisted (prevalence fell from 35 to 8 %). From June to October 2012 (rainy season) the number of clinical episodes of *P. falciparum* was six times lower (46), than during the same period in the previous year (290).

**Conclusion:**

Mass drug administration with dihydroartemisinin-piperaquine may be an effective strategy to eliminate *P. falciparum* rapidly where multi-drug resistance is present.

## Background

The emergence and spread from Asia to Africa of first chloroquine resistance and then anti-folate resistance in *P. falciparum* led to the loss of millions of lives. Relative resistance to artemisinin derivatives has emerged recently in Western Cambodia making it imperative to eliminate these parasites rapidly to prevent their spread across Asia to Africa and another disastrous upsurge in malaria mortality. Although this apparently has now the highest priority for malaria control programmes [1], a consensus on how this can be best achieved is yet to be reached. Current strategies and plans provide little detailed guidelines [2, 3] and the continued strengthening of existing malaria control measures has failed to prevent the westward spread of artemisinin resistant parasites [4]. Treating symptomatic patients and preventing transmission only in these areas cannot achieve malaria elimination rapidly enough. This is because within these areas there are apparently healthy individuals who have asymptomatic malaria parasitaemia who sustain malaria over the dry season and act as a reservoir of transmission. The epidemiology of malaria transmission in low-endemic areas is still poorly understood [5, 6], and current estimates of the true burden might be very inaccurate [7].

One potential approach recently advocated by the WHO is to screen individuals in endemic areas for malaria and treat those who are positive either by microscopy, rapid tests, or by PCR (Focal Screen and Treat; FSAT) [8]. This approach assumes that most infections will be detected by such methods. The PCR methods used are more sensitive than microscopy but, as the blood sample volumes assayed are typically small (a few microlitres), they have detection limits of approximately 1,000 parasites/mL of blood. Individuals with parasite densities below this remain undetected and untreated. An alternative more radical approach, which would treat effectively all parasitaemic individuals, is to treat the entire population in the area, a strategy applied in different ways over the past 100 years with varying results [9–12]. However recent experience with this approach is scarce.

In response to requests for assistance because of reportedly large numbers of malaria cases occurring in remote villages adjacent to the border with Thailand, an area where multi-drug resistant *P. falciparum* is prevalent an urgent assessment was conducted. Cross sectional surveys were conducted in three of the affected villages using a highly sensitive PCR detection method [7]. The results were presented to the communities and different containment strategies discussed. Village malaria workers were trained in detection and treatment. Malaria chemoprevention was endorsed by the residents of one village where this was possible, and its impact was assessed.

## Methods

Along the Thailand-Myanmar border the transmission of malaria is low and seasonal [5, 13, 14]. Most clinical cases are seen between May and December. Incidence rises abruptly with the onset of the rains in April–May each year, potentially reaching epidemic proportions and then declines at the height of the rainy season (August–October) and then sometimes peaks again at the beginning of the cool season (November–December). Approximately half the cases are *P. vivax* and the other half *P. falciparum. Plasmodium malariae* and *P. ovale* infections are reported rarely. Entomological inoculation rates for the two main species are typically below 2/person/year. The Shoklo Malaria Research Unit has conducted clinical and epidemiological research on malaria on the North Western border of Thailand for the past 28 years and supported regional malaria control efforts, during which time the overall incidence of malaria has declined markedly, the relative proportion of *P. vivax* has increased, and resistance to mefloquine, and now to artemisinin has emerged [15].

Community leaders and key workers were consulted about the project, and approval was obtained from the Tak Province Community Ethics Advisory Board (T-CAB) [16]. The T-CAB which consist of representatives from both the local ethnic Karen and Burman border communities has been the primary ethics review body for health care interventions along the border for the past 6 years [17].

Surveys were conducted in the three villages (PLU, WLM and TMK) located on the Thailand-Myanmar border and the need to screen everyone for malaria parasitaemia within a short time in the surveys was agreed. Blood samples (>1 mL from adults and 0.5 mL from children older than 5 years) were then taken from the participants for the qPCR assay. Children 5 years old or younger were not sampled. Standard malaria thick and thin smears were prepared and stained with Giemsa for all participants. Samples were taken into EDTA tubes transported on ice and then stored at −80 °C. All subjects were examined clinically and axillary temperatures recorded. If patients were ill a malaria rapid test was performed and appropriate treatment was given.

### Measurement of parasitaemia

Standard microscopy was performed on Giemsa-stained thick films and the number of parasites per 500 white blood cells was counted. The sensitive high volume PCR assay has been described in detailed elsewhere [7]. Briefly, the DNA template for PCR detection and quantification of *Plasmodium* was purified from the thawed packed red blood cells samples. The volume of each sample was carefully measured before DNA extraction, which was undertaken using a QIAgen Blood Mini kit^®^ for sample volumes ≤200 µL (equivalent to 400 µL of whole blood assuming a normal haematocrit of 50 %), or a QIAgen Blood or Midi kit^®^ for sample volumes between 200 and 2,000 µL packed red blood cells (Qiagen, Germany). The purified DNA was dried in a centrifugal vacuum concentrator and then suspended in a small volume of PCR grade water so as to concentrate it. The PCR sensitivity had a lower limit of detection of only 22 parasites per 1,000 µL of whole blood. For samples where the qPCR was positive an attempt was made to determine the *Plasmodium* species present using nested PCR protocols specific to *P. falciparum,P. vivax* and *P. malariae* as described previously [18–20]. Samples for which there was insufficient DNA to do this, or where no amplification was obtained were reported as being of indeterminate species.

### Provision of malaria chemoprevention

When the results of the initial surveys became available they indicated very high rates of asymptomatic malaria parasite carriage in all three villages (see below). After urgent validation and review of the PCR results and subsequent consultation and discussion with community leaders and the T-CAB, by April 2012 it was decided mutually that the best approach to reducing the malaria burden in the imminent malaria season was to strengthen local diagnosis and treatment of malaria and if possible to offer urgent supervised chemoprevention to the whole community, excluding children under 14 and pregnant women. It was agreed that if chemoprevention was provided then it would be necessary to monitor its acceptability and effects. However two of the three villages were inaccessible during the rainy season and so chemoprevention could not be deployed without the necessary supervision. In the closest village (WLM), which could be reached, a full 3-day treatment course of dihydroartemisinin-piperaquine (7.5/60 mg/kg) was provided to all non-pregnant participants (age 14 years and above) at monthly intervals for 3 months (June, July, August 2012) and a follow up malaria survey was performed in December 2012 to assess impact. These plans were discussed with and approved by the community and individuals who did not wish to participate were free to do so without affecting their access to health care and malaria treatments.

## Results

In the 5 years before the three surveys presented here, the prevalence of *P. falciparum* and *P. vivax* malaria by microscopy in 6,943 persons in 12 villages in this region ranged from 1.3 to 7 % and 8.1 to 9.7 %, respectively.

The initial surveys presented here were conducted in May 2010 (PLU), December 2011 (WLM) and March 2012 (TMK). The entire population present in each village was invited to participate. In total 580 people were screened in PLU, 536 in WLM, and 401 in TMK (Table 1). This represented 580/634 (91.5 %), 536/674 (79.5 %), and 401/678 (59.1 %) of the total village populations respectively, of whom 356 (PLU), 385 (WLM) and 286 (TMK) provided a blood sample for qPCR.

**Table 1:** Participants in the three villages

	Village 1 (PLU) May 2010	Village 2 (WLM) Dec 2011	Village 3 (TMK) May 2012
Number of participants (% of total in population)			
Adult males	177/193 (92 %)	177/190 (93 %)	75/160 (47 %)
Adult females	169/186 (91 %)	146/201 (73 %)	90/145 (62 %)
Children	234/255 (92 %)	213/283 (75 %)	236/373 (63 %)
Total	580/634 (91 %)	536/674 (80 %)	401/678 (60 %)

**Fig. 1:**
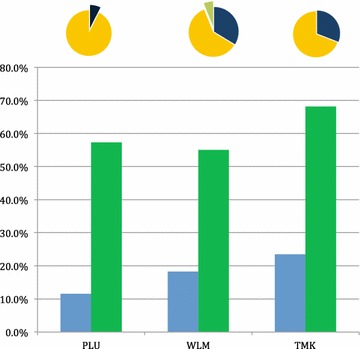
Prevalence of malaria by species in the three villages. The *upper panel* shows the distribution of the species: *black*: *P. falciparum*, *orang*e: *P. vivax*, *green*: mixed infections. The *lower panel* shows the prevalence of malaria in the three villages. *Blue*: microscopy, and *Green*: qPCR.

Overall 11.6 % (67/577), 18.3 % (98/536), and 23.4 % (94/401) of the villagers were malaria parasite positive by microscopy in PLU, WLM and TMK, respectively (Fig. 1; Table 2). The corresponding figures for the testing by qPCR were 2–5 times higher: 204/356 (57.3 %), 212/385 (55.1 %) and 195/286 (68.2 %), respectively (Table 3).

**Table 2:** Prevalence in the three villages by microscopy

	Village 1 (PLU) May 2010	Village 2 (WLM) Dec 2011	Village 3 (TMK) May 2012
Number (%) positive for P. falciparum*			
Adult males	2/176 (1.2 %)	22/177 (12.4 %)	6/75 (8.0 %)
Adult females	3/167 (1.7 %)	2/146 (1.4 %)	7/90 (7.8 %)
Children	0/234 (0 %)	15/213 (7.0 %)	28/236 (11.9 %)
Total	5/577** (0.9 %)	39/536 (7.3 %)	41/401 (10.2 %)
Number (%) positive for *P. vivax* ^*^			
Adult males	29/176 (16.5 %)	16/177 (9.0 %)	13/75 (17.3 %)
Adult females	17*/167 (10.2 %)	12/146 (8.2 %)	9/90 (10.0 %)
Children	16/234 (6.8 %)	33/213 (15.5 %)	63/236 (26.7 %)
Total	62/577* (10.7 %)	61/536 (11.4 %)	85/401 (21.2 %)

* Mixed infections are counted in both species.

** 3 slides were unreadable.

**Table 3:** Malaria species by sensitive PCR in the three villages

	Village 1 (PLU) May 2010	Village 2 (WLM) Dec 2011	Village 3 (TMK) May 2012
Number (%) positive for P. falciparum*			
Adult males	21/148 (14.2 %)	42/171 (24.6 %)	21/71 (29.6 %)
Adult females	18/127 (14.2 %)	15/130 (11.5 %)	18/90 (20.0 %)
Children	19/81 (23.5 %)	14/84 (16.7 %)	31/125 (24.8 %)
Total	58/356 (16.3 %)	80/385 (18.4 %)	71/286 (24.5 %)
Number (%) positive for P. vivax*			
Adult males	63/148 (42.5 %)	56/171 (32.7 %)	31/71 (43.7 %)
Adult females	39/127 (30.7 %)	40/130 (30.8 %)	32/90 (35.6 %)
Children	20/81 (24.7 %)	46/84 (54.8 %)	71/125 (56.8 %)
Total	122/356 (34.3 %)	124/385 (36.9 %)	134/286 (46.9 %)

* Parasites could not be identified in 40/356 (12.4 %) and 30/385 (7.8 %) and 26/286 (9.1 %) positive samples in villages 1, 2 and 3 respectively. Mixed infections are counted in both species.

In total 611 infections were detected, but for 100 of these the parasite species could not be determined because of insufficient DNA. Of the remaining 511 infections, approximately one-third were *P. falciparum* and two-thirds were *P. vivax*. One *P. malariae* infection was identified and 86 (14.1 %) were mixed (*P. falciparum* and *P. vivax*) infections. Overall the parasitaemias were low and 30.1 % (184/611) of the positive qPCR samples had parasite counts below 1,000 per mL and 4.1 % (25/611) had parasite densities below 100 per mL of blood **(**Table 4). Overall 72.3 % (442/611) of the infections detected by qPCR had a density below 50 per microlitre (the lower limit of detection by microscopy). Of the 74 samples that were positive for *P. falciparum* that were successfully sequenced for the *k*-*13* gene, 46 (62 %) were mutant.

**Table 4:** Geometric Mean of parasitaemia by microscopy and PCR in the three villages

	Village 1 (PLU) May 2010		Village 2 (WLM) Dec 2011		Village 3 (TMK) May 2012	
	Microscopy	PCR	Microscopy	PCR	Microscopy	PCR
Geometric mean (range) all species	63.0 (32–256)	7.82 (0.28–186,948)	109 (16–5,888)	50.31 (0.25–751,510)	85.0 (2–4,224)	26.07 (0.11–296,713)
Geometric mean (range) *P. falciparum*	170.0 (90–256)	2.95 (0.34–5,999)	110 (16–5,792)	69.52 (0.27–751,510)	96.0 (16–4,224)	79.01 (0.25–296,713)
Geometric mean (range) *P. vivax*	47.9 (32–144)	24.38 (0.31–25,136)	109 (16–5,888)	36.78 (0.3–108,318)	79 (2–2,880)	14.79 (0.11–97,719)

### After chemoprevention

In June 2012, 562 villagers in WLM participated to the intervention of whom 346 provided a qPCR blood sample and 206 (36.7 %) received a treatment dose of DP chemoprevention. This was repeated in the following 2 months and the corresponding numbers were 416, 270 and 161 (38.7 %) in July and 263, 178 and 114 (43.3 %) in August 2012. A total 217 inhabitants received at least one dose of DP chemoprevention, of whom 98 (45.2 %) received the complete three treatment doses and 155/217 (71.4 %) participants received two consecutive months of chemoprevention. Those who did not receive the complete course of chemoprevention either refused or had left the village. In December 2012 426 villagers participated in the final survey of whom 375 provided a sample for qPCR. The treatments were well tolerated with no serious adverse events reported. A few participants reported mild dizziness following treatment.

**Fig. 2:**
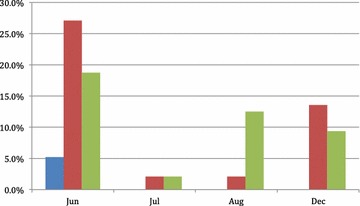
Prevalence of malaria in the 96 participants who received the full course of DP. *Blue*: *P. falciparum*, *Red*: *P. vivax*, *Green*: indeterminate species.

The results of the post-chemoprevention surveys conducted in June, July, August, and December 2012 in WLM indicated an overall reduction in the prevalence of malaria in the population (Table 5). In July, one of the five *P. falciparum* cases was in a participant who had received DP in June and who was negative by PCR in August and December. The remaining *P. falciparum* cases (four in July and five in August) were all in people who had not previously received DP. Eight of the participants to the June 2012 survey were already positive by qPCR in the December 2011 survey (i.e. 6 months earlier), five with mixed infections and three with *P. falciparum*. Of those with mixed infections in December 2011, two had *P. vivax*, two mixed infections and one *P. falciparum* in the June 2012 survey. Of the three remaining *P. falciparum* infections of December 2011, two had *P. vivax* and one was negative in June 2012 (Table 6). In the sub-group of 96 people (83 adults and 13 children) who received the three courses of DP and also provided a venous sample at each survey, the proportion of *P. falciparum* infections declined from 5.2 % (5/96) in June to nil in July, August and December while the proportion of *P. vivax* infections declined from 27.1 % (26/96) to 2.1 % (8/96) in July, 2.1 % (8/96) in August and was 13.5 % (13/96) in December (Fig. 2). The plasmodial species could not be determined in 18.8 % (18/96) in June, 8.3 % (8/96) in July, 12.5 % (12/96) in August and 9.4 % (9/96) in December 2012. During the period from June to October 2012 (rainy season) the number of clinical episodes of *P. falciparum* detected by the malaria post was over six times lower (46) than in the same period of the previous year (290).

**Table 5:** Malaria prevalence by Microscopy and PCR post-chemoprevention in WLM

	June 2012		July 2012		August 2012		December 2012	
	Number (%) of M/S*	Number (%) of PCR	Number (%) of M/S**	Number (%) of PCR	Number (%) of M/S	Number (%) of PCR	Number (%) of M/S***	Number (%) of PCR
*P. falciparum*	8/560* (1.4 %)	24/346 (6.9 %)	0/409** (0.0 %)	5/270 (1.9 %)	1/263 (0.4 %)	5/178 (2.8 %)	1/425*** (0.2 %)	1/370 (0 %)
Adult male	4/151 (2.6 %)	8/72 (11.1 %)	0/112 (0.0 %)	1/60 (1.6 %)	1/76 (1.3 %)	3/42 (7.1 %)	1/93 (1.1 %)	0/92 (0.0 %)
Adult female	1/171 (0.6 %)	8/121 (6.6 %)	0/117 (0.0 %)	0/96 (0.0 %)	0/75 (0.0 %)	1/58 (1.7 %)	0/134 (0.0 %)	0/133 (0.0 %)
Child	3/238 (1.3 %)	8/153 (5.2 %)	0/180 (0.0 %)	4/114 (3.5 %)	0/112 (0.0 %)	1/78 (1.3 %)	0/198 (0.0 %)	0/145 (0.0 %)
*P. vivax*	61/560 (10.9 %)	122/346 (35.5 %)	18/409 (4.4 %)	45/270 (16.7 %)	14/263 (5.3 %)	30/178 (16.9 %)	37/425 (8.7 %)	29/370 (7.8 %)
Adult male	16/151 (10.6 %)	23/72 (31.9 %)	1/112 (0.9 %)	4/60 (6.7 %)	2/76 (2.6 %)	3/42 (7.1 %)	7/93 (7.5 %)	4/92 (4.3 %)
Adult female	9/171 (5.3 %)	35/121 (28.9 %)	1/117 (0.9 %)	2/96 (2.1 %)	1/75 (1.3 %)	2/58 (3.4 %)	7/134 (5.2 %)	7/133 (5.3 %)
Child	36/238 (15.1 %)	64/153 (41.8 %)	16/180 (8.9 %)	39/114 (34.1 %)	11/112 (9.8 %)	25/78 (32.0 %)	23/198 (11.6 %)	18/145 (12.4 %)
Indeterminate	NA	57/346 (16.5 %)	NA	27/270 (10.0 %)	NA	27/178 (15.2 %)	NA	15/370 (4.1 %)
Adult male	NA	12/72 (16.7 %)	NA	7/60 (11.7 %)	NA	11/42 (26.2 %)	NA	4/92 (4.3 %)
Adult female	NA	23/121 (19.0 %)	NA	6/96 (6.3 %)	NA	5/58 (8.6 %)	NA	0/133 (0.0 %)
Child	NA	22/153 (14.4 %)	NA	14/114 (12.3 %)	NA	11/78 (14.1 %)	NA	6/145 (4.1 %)
Negative	493/560 (88.0 %)	153/346 (44.2 %)	391/409 (95.6 %)	196/270 (72.6 %)	248/263 (94.3 %)	117/178 (65.7 %)	387/425 (91.1 %)	326/370 (88.1 %)
Adult male	131/151 (86.8 %)	32/72 (44.4 %)	111/112 (99.1 %)	48/60 (80.0 %)	73/76 (96.1 %)	25/42 (59.5 %)	85/93 (91.4 %)	84/92 (91.3 %)
Adult female	111/171 (94.7 %)	56/121 (46.2 %)	116/117 (99.1 %)	88/96 (91.7 %)	74/75 (98.7 %)	50/58 (86.2 %)	127/134 (94.8 %)	126/133 (94.7 %)
Child	73/238 (84.0 %)	65/153 (42.5 %)	164/180 (91.1 %)	60/114 (52.6 %)	104/112 (90.2 %)	42/78 (53.8 %)	175/198 (88.4 %)	121/145 (83.4 %)

Mixed infections are counted in both species.

* 2 slides were unreadable.

** 7 slides were unreadable.

*** 1 slide was unreadable.

**Table 6:** Details of eight participants positive by qPCR in December 2011

Case	Dec 2011	June 2012		July 2012		August 2012		Dec 2012
	PCR result	MDA	PCR result	MDA	PCR result	MDA	PCR result	PCR result
1	PF	Y	PF	N	N	N	N	Neg
2	PF/PV	N	PF/PV	N	PF/PV	N	N	Neg
3	PF	Y	PF	Y	Neg	N	N	Neg
4	PF/PV	N	PV	N	Neg	N	Neg	PV
5	PF	Y	NEG	Y	Neg	N	N	Neg
6	PF/PV	N	PF/PV	N	PF/PV	N	Indeterminate	PV
7	PF/PV	N	PF	N	N	Y	PV	Neg
8	PF/PV	Y	PV	Y	PV	N	N	Neg

## Discussion

Malaria has been substantially underestimated in this area, as in other areas in this region with similar epidemiology [7]. A high proportion of apparently healthy individuals living in an area conventionally classified as having “hypoendemic” malaria are parasitaemic. This has profound implications for malaria control and elimination efforts, and in particular for the urgent need to eliminate artemisinin resistant falciparum malaria. It is clear that strengthening treatment facilities (passive detection) or conventional active detection (FSAT) will identify only some of the parasitaemic individuals, and so will not eliminate malaria rapidly.

The experience reported here is of a malaria survey and intervention conducted in response to a request for assistance in an area where malaria epidemics are not uncommon. It was not a carefully designed prospective study so it has certain limitations, notably the low coverage and short duration of follow up. Nevertheless the information gained was of value both for the understanding of malaria epidemiology and also the design and evaluation of control interventions. The majority of asymptomatic malaria parasite carriers in this area of low seasonal malaria transmission had parasite densities below those detectable by microscopy and many were below the limit of detection of the commonly used low volume (5 µL) PCR methods. It seems likely that even the improved detection method with a limit of detection of approximately 20 parasites/mL (corresponding to a total of 100,000 parasites in the blood of an adult), might still not capture all carriers. This level of sensitivity may also detect infections as they emerge from the liver stage development, as was observed nearly 70 years ago in large blood volume sub-inoculation experiments [21], that might self-cure. At very low parasite densities, false PCR positivity is clearly a major concern for epidemiological assessments. The consistent negativity of control samples provides reassurance that the higher prevalence estimates are not inflated significantly. The observation that the individuals with very low parasite densities became negative by qPCR after treatment provides further support that these were active low-density infections and not simply artefacts. The need for a relatively large amount of venous blood (1–2 mL in adults) for the qPCR assay and the requirement to process the sample within 24 h are significant operational obstacles. This detection method should not be seen as a diagnostic tool for case detection (it is too sensitive), but as a method of detecting asymptomatic malaria and thereby assessing the size of the sub-microscopic reservoir in the community. Estimates can be achieved by sampling a small number of randomly selected adults. The possibility that the true prevalence of malaria in these villages is even higher cannot be excluded. The high prevalence of sub-microscopic malaria was little influenced by seasonality whereas most symptomatic malaria occurs during the rainy season. Similar observations have been made in higher transmission settings where symptomatic malaria often coincides with the rains but asymptomatic carriage is much less affected by season. The prevalence of asymptomatic *P. vivax* was approximately twice that of *P. falciparum*, and their density profiles were approximately similar. The proportion of *P. falciparum* to *P. vivax* in asymptomatic carriers was similar to that seen in symptomatic patients in malaria clinics.

The discovery of a substantial number of parasite positive asymptomatic individuals was discussed with the affected village representatives and a joint decision taken to try and reduce this previously undetected burden of malaria. Malaria diagnosis and treatment were strengthened and three rounds of chemoprevention was offered to the whole community for the coming rainy season, though for logistical and security reasons this was only possible for one of the villages. The prevalence of *P. falciparum* malaria by microscopy and by qPCR declined sharply after the intervention and was still very low 6 months later, while the prevalence of *P. vivax* infections declined initially but rose again. This is most likely due to relapses from liver stages. There is increasing evidence that *P. vivax* hypnozoites can remain dormant for long periods even in tropical areas. The elimination of hypnozoites would require long courses of primaquine, a drug associated with a significant risk of haemolysis in this population where the prevalence of G6PD deficiency is high. There were few clinical episodes of malaria detected during the rainy season following the intervention. However less than half of the entire population received the full three doses of DP, so the overall impact on malaria transmission was sub-optimal. This underlines the crucial importance of an effective community engagement process in this type of intervention. Another significant limitation of this survey is the exclusion of young children and pregnant women. Obviously, if elimination of *P. falciparum* malaria is the goal, these two vulnerable groups will have to be accommodated. Studies are on going to address this important question.

From an operational perspective, screen and treat approaches to malaria elimination are difficult, insufficiently effective, and costly. The only way to eliminate malaria rapidly in these populations is to treat the entire population with effective anti-malarial drugs. How, when and how often this should be done needs urgent investigation. Whether such an approach will be feasible and effective is not known. This region is in a race against time to contain and eliminate artemisinin resistance but the challenges are formidable. As shown in this experience, access to communities can be difficult because of the terrain and because of political instability. In the 2 years that follow this intervention, a more detailed prospectively designed project was undertaken in four new villages with the participation of local Non Governmental Organizations and Community Based Organisations. This ensured accessibility and participation in this politically fragmented population.
